# Comparison of hamstring and quadriceps strength after anatomical versus non-anatomical anterior cruciate ligament reconstruction: a retrospective cohort study

**DOI:** 10.1186/s12891-021-04350-1

**Published:** 2021-05-18

**Authors:** Hai Jiang, Lei Zhang, Rui-Ying Zhang, Qiu-Jian Zheng, Meng-Yuan Li

**Affiliations:** 1 Department of Orthopedic Surgery, Guangdong Provincial People’s Hospital, Guangdong Academy of Medical Sciences, NO. 106, Zhongshan 2nd Road, 510000 Guangzhou, China; 2 Department of Rehabilitation, Guangdong Provincial People’s Hospital, Guangdong Academy of Medical Sciences, NO. 106, Zhongshan 2nd Road, 510000 Guangzhou, China

**Keywords:** Hamstring and quadriceps strength, Anatomical, Non-anatomical, Anterior cruciate ligament reconstruction

## Abstract

**Background:**

Strength recovery of injured knee is an important parameter for patients who want to return to sport after anterior cruciate ligament reconstruction (ACLR). Comparison of muscle strength between anatomical and non-anatomical ACLR has not been reported.

**Purpose:**

To evaluate the difference between anatomical and non-anatomical single-bundle ACLR in hamstring and quadriceps strength and clinical outcomes.

**Methods:**

Patients received unilateral primary single-bundle hamstring ACLR between January 2017 to January 2018 were recruited in this study. Patients were divided into anatomical reconstruction group (AR group) and non-anatomical reconstruction group (NAR group) according to femoral tunnel aperture position. The hamstring and quadriceps isokinetic strength including peak extension torque, peak flexion torque and H/Q ratio were measured at an angular velocity of 180°/s and 60°/s using an isokinetic dynamometer. The isometric extension and flexion torques were also measured. Hamstring and quadriceps strength were measured preoperatively and at 3, 6, and 12 months after surgery. Knee stability including Lachman test, pivot-shift test, and KT-1000 measurement and subjective knee function including International Knee Documentation Committee (IKDC) and Lysholm scores were evaluated during the follow-up.

**Results:**

Seventy-two patients with an average follow-up of 30.4 months (range, 24–35 months) were included in this study. Thirty-three were in AR group and 39 in NAR group. The peak knee flexion torque was significant higher in AR group at 180°/s and 60°/s (*P* < 0.05 for both velocity) at 6 months postoperatively and showed no difference between the two groups at 12 months postoperatively. The isometric knee extension torque was significant higher in AR group at 6 months postoperatively (*P* < 0.05) and showed no difference between the two groups at 12 months postoperatively. No significant differences between AR group and NAR group were found regarding knee stability and subjective knee function evaluations at follow-up.

**Conclusions:**

Compared with non-anatomical ACLR, anatomical ACLR showed a better recovery of hamstring and quadriceps strength at 6 months postoperatively. However, the discrepancy on hamstring and quadriceps strength between the two groups vanished at 1 year postoperatively.

## Introduction

Anterior cruciate ligament (ACL) is the most commonly injured ligament of the knee [1]. ACL injuries interrupt the normal kinematics of knees with or without meniscal tears. After ACL injury, the knee joint remains unstable and becomes more prone to further injuries including meniscal and articular cartilage injuries which may lead to osteoarthritis [2–4]. The ACL reconstruction (ACLR) is considered as the standard treatment for those patients who expected a restoration of knee function.

Considering the position of femoral tunnel, transtibial (TT) technique is a traditional method, which is believed to make the tunnel aperture far away the anatomical footprint of ACL. Currently, more surgeons prefer to use anteromedial (AM) portal technique to drill femoral tunnel [5], regarding it lead to an anatomical ACLR [6, 7]. However, as the restriction of surgical equipment and anatomic variation of patients, some of them received non-anatomical ACLR with AM technique. Many studies have reported that anatomical ACLR can restore better rotational stability and clinical outcomes than non-anatomical ACLR [8–14]. Muscle strength deficit after ACLR have been demonstrated to decrease stability and force attenuation for up to 2 years, and this may lead a high risk in future knee injury [15]. Therefore, there might be a better muscle strength after anatomical ACLR. However, to our knowledge, comparison of muscle strength has not been reported in a comparative study involving anatomical and non-anatomical ACLR.

Strength recovery of injured knee is an important parameter for patients who want to return to sport after ACLR [16, 17]. Isokinetic dynamometry is considered the ‘gold standard’ for measuring muscle strength and is commonly applied as part of criteria for return to sport in previous studies [18, 19]. Torques are frequently measured in isokinetic conditions as this is a traditional method of muscle strength assessment [20, 21]. Isokinetic and isometric tests could also monitor biomechanical strength and strength-speed characteristics of the muscles affecting the operated knee joint[22]. Several studies researched the efficiency of the injured knee with the use of isokinetic and isometric tests by analyzing mean extension and flexion peak torques [22–25].

The main purpose of the current study was to evaluate the difference between anatomical and non-anatomical single-bundle ACLR in terms of hamstring and quadriceps strength and clinical outcomes. We hypothesized that anatomical ACLR would be associated with better strength of hamstring and quadriceps and clinical outcomes than non-anatomical ACLR.

## Methods

 This retrospective cohort study was approved by the ethics committee of Guangdong Provincial People’s Hospital (No. GDREC2019639H) and was performed in strict accordance with the ethical standards stipulated in the 1964 Declaration of Helsinki and its later amendments. Written informed consent was obtained from all patients before enrollment.

Patients with ACL injuries who were treated at our institution between January 2017 to January 2018 were recruited in this study. The inclusion criteria included unilateral primary single-bundle hamstring ACLR with closed epiphyseal plate. The exclusion criteria included sever restricted range of motion that cannot finish preoperative measurement, multiligament injuries, revised ACLR, bilateral injuries and lost to follow-up.

### Surgical procedure

All surgical procedures were performed by a senior surgeon (Q.J.Z.). Patients were given lumbar anesthesia. The anteromedial (AM) and anterolateral (AL) portal were made routinely. The arthroscopic exploration and debridement were performed firstly. Meniscal tear and cartilage lesion were diagnosed and treated if existing. Meniscal suture was performed if tear was located in red zone, otherwise partial resection was performed. Microfracture was performed if cartilage lesion was grade III or IV via Outerbridge classification [26]. The semitendinosus and gracilis tendons were harvested and knitted as quadrupled grafts for ACLR. The femoral tunnel aperture and tibial tunnel aperture were created separately. A femoral drill guide was placed at the center of femoral footprint through the AM portal with the knee flexed to 120°. The tibial tunnel was located based on the ACL anatomical tibial footprint and drilled with use of tibial tunnel guide. The grafts were fixated with cortical button on femoral side and interference screw on tibial side, at knee flexion angle of 30° and initial tension of 80 N. The wounds would be closed if the knee stability and graft tension met the surgeon’s satisfaction.

### Postoperative rehabilitation

The elastic bandage was applied immediately after surgery, for alleviating knee swelling and pain. Knee brace was used to protect the knee for 12 weeks [27]. Patients were permitted with partial weight bearing as early as possible, except those with meniscal sutures allowed non-weight bearing with crutches for 4 weeks. The non-weight bearing knee flexion exercise was performed to improve the range of motion from the second week. Within 4 weeks, patients were encouraged to perform ankle pump exercise, isometric quadriceps and hamstring contractions, straight and side leg raising exercises. Full weight-bearing exercise was permitted from 6 weeks after surgery. Running and swimming was permitted until 3 months, but contact sports were not suggested until 12 months after operation [27, 28].

### Tunnel placement determination

**Fig. 1:**
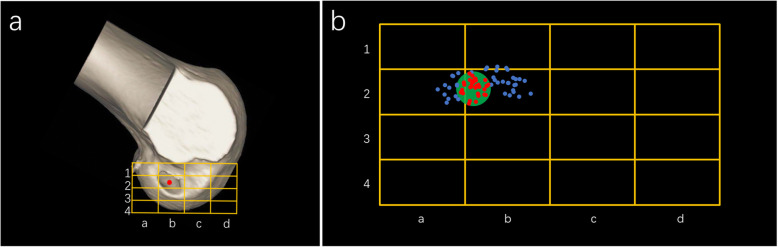
The measurement of femoral tunnel aperture. **a** The center (red point) of femoral tunnel aperture was measured by Bernard quadrant method (4 × 4 grid). **b** The green circle represented the standard area of anatomical femoral footprint center described by Xu et al.*. The red points within the green circle represented the anatomical femoral reconstruction, the blue points outside the green circle represented the non-anatomical femoral reconstruction

All patients were scanned by a CT scanner in the supine position with knees extended and thighs horizontal and parallel on the second day after the surgery. The 3D model of distal femur and proximal tibia were reconstructed on PACS system. The femoral model was cut off at sagittal plane along the highest point of intercondylar notch and medial condyle was removed, and then the model was rotated to show the medial side of lateral condyle. The Bernard quadrant method was used to measure the femoral tunnel position (Fig. 1). This method consists of 4 distances, including total diameter of lateral condyle along Blumensaat’s line (distance t), maximum intercondylar notch height (distance h), distance from center of footprint to proximal border (distance x), and distance from center of footprint to Blumensaat’s line (distance y). The centers of femoral tunnel were recorded in the shadow/deep direction (distance x/t) in the high/low direction (distance y/h). The distribution of the centers of femoral tunnel aperture was displayed in Fig. 1. Xu et al. [29] described the standard area of anatomical femoral footprint center was 27.53 % ± 4.58 %, 35.85 % ± 9.2 % (x, y) of the ACL as a whole bundle. In this study, the centers of femoral tunnel aperture within the standard area were defined as anatomical reconstruction, while the centers outside the standard area were defined as non-anatomical reconstruction. Therefore, the enrolled patients were divided into anatomical reconstruction (AR) group and non-anatomical (NAR) group according to the location of femoral tunnel. The anteroposterior and lateral-medial positions of tibial tunnel aperture were measured. The centers of tibial tunnel were recorded in anteroposterior position and lateral-medial position. The measurement was performed by an orthopedic surgeon (M.Y.L.) who was blinded to outcomes of the patients.

### Measurement of hamstring and quadriceps strength

Hamstring and quadriceps strength were measured preoperatively and at 3, 6, and 12 months after surgery. The contralateral leg was tested first, and then injured leg. The hamstring and quadriceps isokinetic strength were measured in the seated position at an angular velocity of 180°/s and 60°/s using an isokinetic dynamometer (IsoMed 2000, D&R GmbH, Hemau, Germany). The subjects performed a 5-minute warm-up on the dynamometer before the measurements were conducted. Ten duplicate leg extension and flexion measurements between knee joint angle of 10° to 90° were performed with adequate rest periods during the interval. The peak extension torque, peak flexion torque and H/Q ratio were recorded for further analysis. The isometric extension and flexion torques were measured as well, by patients making their most effort to perform knee flexion and extension with knee joint fixed at 90°. Limb symmetry index (LSI) was recorded as percentage of the surgical limb over the nonsurgical limb at the final follow-up.

### Clinical evaluation

Lachman test, pivot-shift test, and KT-1000 arthrometer (MEDmetric Corp, San Diego, CA, USA) with an anterior tibial translational force of 89 N were used to evaluate postoperative knee stability [30, 31]. International Knee Documentation Committee (IKDC) and Lysholm scores used to assess the subjective knee function were collected simultaneously [26, 32, 33]. IKDC and Lysholm scores were registered between 0 and 100, where a higher score represented a better condition of knee joint. Tegner activity score was recorded as well to assess return to sports [34]. Knee stability and subjective knee function were evaluated before the surgery and at the final follow-up by an orthopedic surgeon (M.Y.L.) who was blinded to the two groups. Complications were recorded during the follow-up.

### Statistical analysis

The SPSS 22.0 software package (IBM Inc. USA) was used for statistical analysis. An a priori power analysis was conducted to compute the sample size. For a power of 0.8 and an alpha value of 0.05, The number of patients required in this study was 29 for each group, and the statistical power was 0.81. Descriptive statistics were reported as mean values and standard deviations. Mann-Whitney U test was used to compare the KT-1000 measurement, subjective IKDC scores, Lysholm scores and parameters of muscle strength between AR and NAR groups. The Chi-square (χ^2^) test was used to compare the results of Lachman and pivot-shift tests between the 2 groups. A level of *P* < 0.05 was set for statistical significance.

## Results

**Fig. 2:**
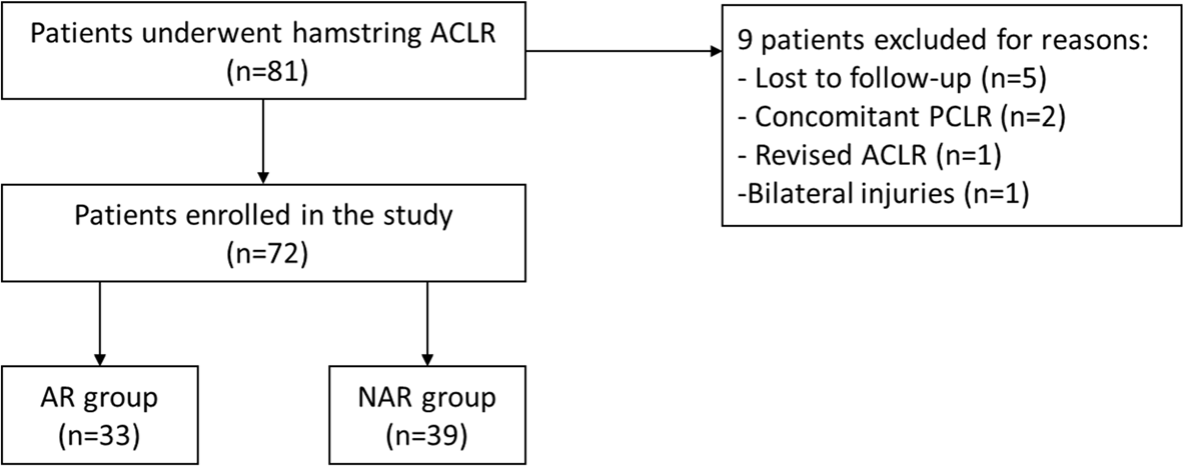
Flow chart of the enrolment of the patients. ACLR, anterior cruciate ligament reconstruction; PCLR, posterior cruciate ligament reconstruction; AR, anatomical reconstruction; NAR, non-anatomical reconstruction

Eighty-one patients underwent hamstring ACLR during the study period. Five patients were lost to follow-up and 2 patients underwent concomitant posterior cruciate ligament reconstruction. One patient received a revised ACLR, and 1 patient had a bilateral injury of knees. Therefore, 72 patients were enrolled in the study. Average follow-up time was 30.4 months (range, 24–35 months). There were 33 patients in AR group and 39 patients in NAR group (Fig. 2). There were no significant differences between the two groups in terms of sex, age, height, weight, BMI, time from injury to surgery and injured side. The initial status of meniscal injuries and treatment of meniscal injuries were also comparable between the two groups (Table 1). No complications were found in the two groups during the follow-up.

**Table 1:** Demographic data and initial surgical findings of two study groups

	AR group	NAR group	*P* value
Sex, male/female, n	29/4	29/10	0.379
Age, y (mean ± SD)	32.0 ± 9.7	32.4 ± 12.7	0.903
Height, cm (mean ± SD)	168.4 ± 7.5	170.1 ± 8.3	0.385
Weight, kg (mean ± SD)	65.2 ± 9.3	70.1 ± 13.4	0.091
BMI (mean ± SD)	22.9 ± 2.5	24.1 ± 3.2	0.102
Injury time, mo (mean ± SD)	6.2 ± 11.3	7.1 ± 12.3	0.452
Injured side, left/right, n	14/19	22/17	0.296
Status, n			0.619
Isolated ACL injuries	5	9	
ACL and medial meniscus injuries	3	5	
ACL and lateral meniscus injuries	12	9	
ACL and both menisci injuries	13	16	
Treatment of meniscal injuries			0.591
Suture	27	28	
Resection	1	2	
Preoperative IKDC score	76.1 ± 19.2	75.4 ± 22.1	0.429
Preoperative Lysholm score	71.5 ± 20.6	69.2 ± 16.9	0.751
Preoperative Tegner score	2.1 ± 1.7	2.4 ± 1.2	0.842
Preoperative knee stability measurement			
Lachman test, stable/I°/II°/III°	0/0/0/33	0/0/0/39	1.000
Pivot-shift test, stable/I°/II°/III°	0/0/0/33	0/0/0/39	1.000
KT-1000 side-to-side difference, mm	5.7 ± 1.7	5.8 ± 2.1	0.864

Descriptive data is presented as mean and standard deviation

*AR* anatomical reconstruction, *NAR* non-anatomical reconstruction, *SD* Standard deviation, *BMI* body mass index, *mo* month(s), *ACL* anterior cruciate ligament, *IKDC* International Knee Documentation Committee

The centers of femoral tunnel in the shadow/deep direction (distance x/t) were located at 27.9 % ± 2.2 % in AR group and 33.4 % ± 6.8 % in NAR group. The centers of femoral tunnel in the high/low direction (distance y/h) were located at 34.8 % ± 4.0 % in AR group and 32.8 % ± 5.4 % in NAR group. The centers of tibial tunnel in anteroposterior position (37.2 % ± 5.6 % vs. 37.1 % ± 5.3 %, *P* = 0.258) and lateral-medial position (35.6 % ± 2.2 % vs. 35.1 % ± 3.1 %, *P* = 0.742) showed no difference between AR and NAR groups. The center of tibial tunnel of all patients were within the anatomical ACL tibial footprint [35].

**Fig. 3:**
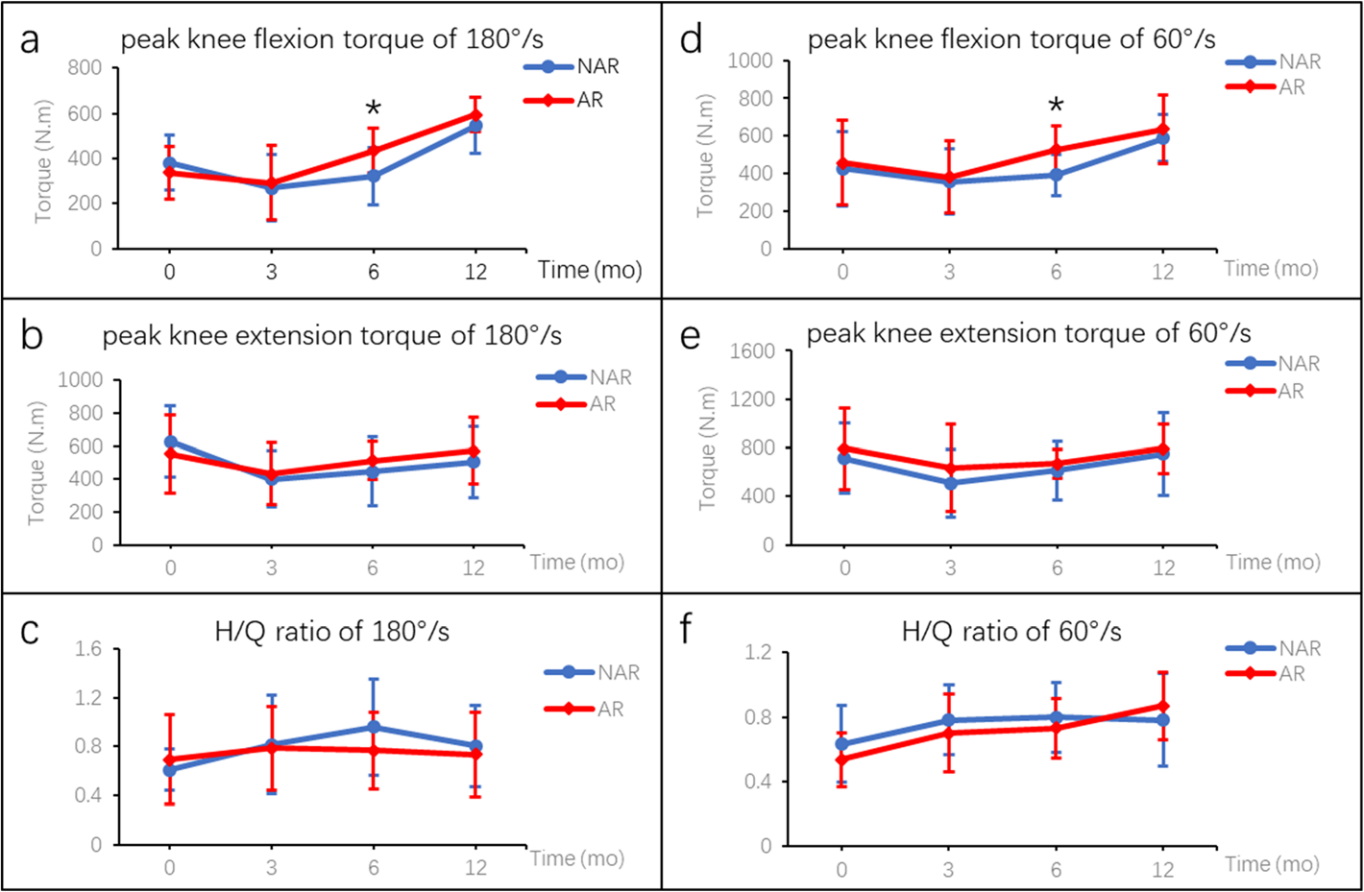
Isokinetic torques (mean ± SD) between AR and NAR groups at preoperation, 3 months, 6 months and 12 months after surgery. **a**, **b** and **c** mean peak knee flexion torque, peak knee extension torque and H/Q ration, respectively, at the velocity of 180°/s. **d**, **e** and **f** mean peak knee flexion torque, peak knee extension torque and H/Q ration, respectively, at the velocity of 60°/s. * indicated *P* < 0.05 between groups. H/Q, hamstring/ quadriceps

**Fig. 4:**
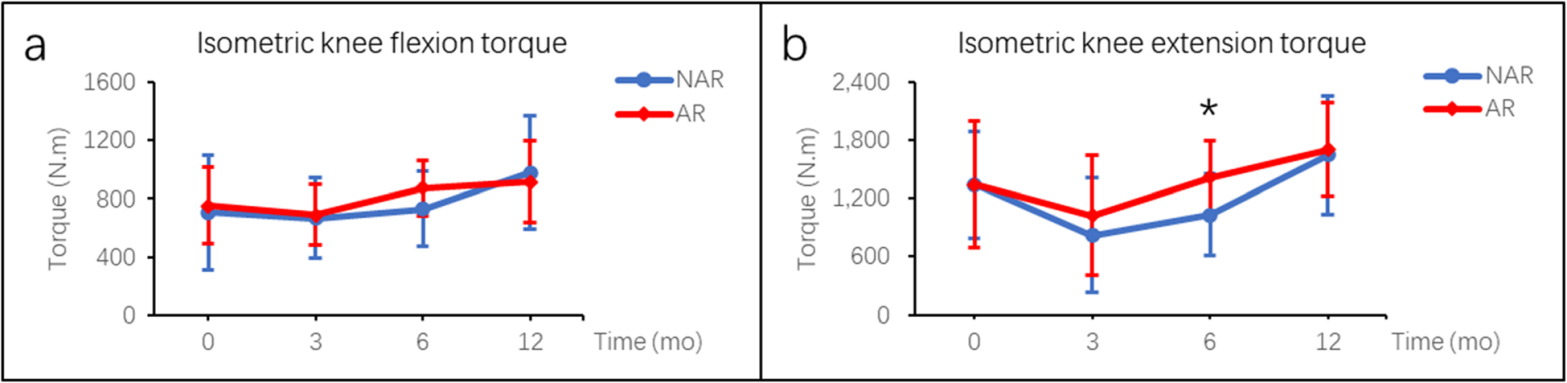
Isometric torques (mean ± SD) between AR and NAR groups at preoperation, 3 months, 6 months and 12 months after surgery. * indicated *P* < 0.05 between groups

**Fig. 5:**
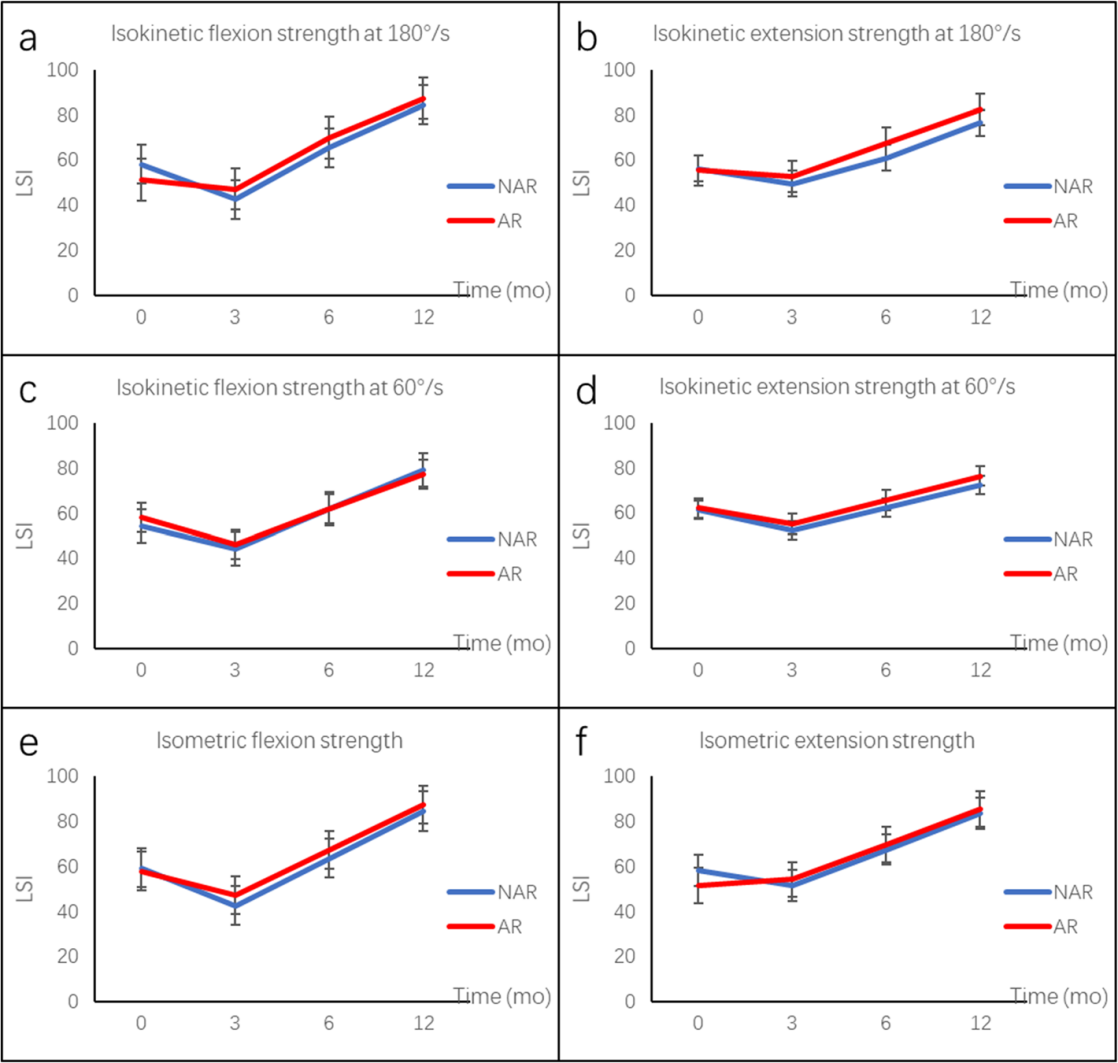
Limb symmetry index between AR and NAR groups at preoperation, 3 months, 6 months and 12 months after surgery. **a** and **b** mean isokinetic flexion and extension strength, respectively, at the velocity of 180°/s. **c** and **d** mean isokinetic flexion and extension strength, respectively, at the velocity of 60°/s. **e** and **f** mean isometric flexion and extension strength, respectively

For hamstring and quadriceps strength analysis, there were no statistical differences between the AR and NAR group in terms of peak knee flexion torque, peak knee extension torque and H/Q ratio at the velocity of either 180°/s or 60°/s (*P* > 0.05) before the surgery. At 12 months after ACLR, all the parameters improved as compared with those before the surgery. The peak knee flexion torque was significant higher in AR group at 180°/s (433.7 ± 99.1 N.m vs. 321.5 ± 127.4 N.m, *P* < 0.05) and 60°/s (528.2 ± 122.4 N.m vs. 392.8 ± 108.6 N.m, *P* < 0.05) at 6 months postoperatively, and showed no significant difference between the two groups at 12 months postoperatively (Fig. 3; Table 2). In the isometric contraction test, the knee extension torque was significant higher in AR group at 6 months postoperatively (1417.7 ± 373.1 N.m vs. 1032.0 ± 424.5 N.m, *P* < 0.05), and showed no significant difference between the two groups at 12 months postoperatively (Fig. 4; Table 2). For LSI measurement, no difference was found between the two groups at preoperation, 3 months, 6 months and 12 months after surgery regarding isokinetic strength or isometric strength (Fig. 5; Table 3).

**Table 2:** Outcomes of isokinetic torques and isometric torques (N.m) between the two groups

		Pre to operation	3 months post-operation	6 months post-operation	12 months post-operation
Isokinetic flexion torque at 180°/s	AR	336.7 ± 118.5	291.1 ± 165.2	433.7 ± 99.1	595.1 ± 76.8
	NAR	380.6 ± 121.8	268.9 ± 146.5	321.5 ± 127.4	545.4 ± 125.1
*P* value		0.612	0.709	**0.037**	0.115
Isokinetic extension torque at 180°/s	AR	553.4 ± 235.7	433.3 ± 189.9	512.1 ± 114.0	572.3 ± 201.7
	NAR	627.6 ± 216.0	400.5 ± 170.2	446.3 ± 208.6	505.2 ± 215.2
*P* value		0.509	0.424	0.271	0.115
H/Q at 180°/s	AR	0.70±0.36	0.78±0.34	0.77±0.31	0.74±0.35
	NAR	0.61±0.17	0.82±0.40	0.96±0.39	0.81±0.33
*P* value		0.651	0.826	0.167	0.425
Isokinetic flexion torque at 60°/s	AR	458.5 ± 225.6	381.0 ± 193.1	528.2 ± 122.4	637.1 ± 182.6
	NAR	426.6 ± 199.4	357.3 ± 171.5	392.8 ± 108.6	588.3 ± 125.7
*P* value		0.700	0.434	**0.013**	0.122
Isokinetic extension torque at 60°/s	AR	795.1 ± 336.4	635.0 ± 361.4	668.4 ± 121.3	794.4 ± 204.9
	NAR	714.2 ± 290.1	509.4 ± 281.6	613.7 ± 239.3	750.4 ± 338.6
*P* value		0.573	0.304	0.441	0.680
H/Q at 60°/s	AR	0.54±0.17	0.70±0.24	0.73±0.18	0.87±0.21
	NAR	0.63±0.24	0.78±0.22	0.80±0.22	0.78±0.29
*P* value		0.473	0.822	0.352	0.475
Isometric flexion torque	AR	754.4 ± 262.7	690.5 ± 207.5	874.5 ± 188.0	914.3 ± 279.0
	NAR	706.4 ± 391.2	665.9 ± 275.5	729.4 ± 259.4	978.3 ± 389.2
*P* value		0.717	0.802	0.035	0.828
Isometric extension torque	AR	1344.8 ± 651.1	1021.1 ± 618.4	1417.7 ± 373.7	1705.3 ± 477.4
	NAR	1341.1 ± 549.8	820.6 ± 592.6	1032.0 ± 424.5	1646.0 ± 616.4
*P* value		0.988	0.198	**0.009**	0.628

Data are reported as mean ± SD

*AR* anatomical reconstruction, *NAR* non-anatomical reconstruction, *H/Q* hamstring/ quadriceps

**Table 3:** Outcomes of muscle strength in LSIs between the two groups

		Pre to operation	3 months post-operation	6 months post-operation	12 months post-operation
Isokinetic flexion strength at 180°/s	AR	51.4 (48.2 to 56.7)	45.4 (39.4 to 52.5)	65.1 (50.9 to 75.8)	84.7 (62.4 to 99.2)
	NAR	58.2 (50.6 to 61.1)	44.6 (40.5 to 56.1)	61.4 (49.5 to 74.1)	78.2 (58.6 to 92.1)
*P* value		0.424	0.365	0.395	0.424
Isokinetic extension strength at 180°/s	AR	55.4 (48.2 to 56.7)	52.8 (42.6 to 62.4)	67.6(50.6 to 78.2)	82.5 (58.6 to 94.1)
	NAR	56.3 (45.6 to 64.1)	49.4 (42.4 to 59.8)	60.9 (47.5 to 77.7)	76.5 (52.7 to 95.6)
*P* value		0.724	0.448	0.321	0.192
Isokinetic flexion strength at 60°/s	AR	58.3 (46.2 to 64.8)	46.3 (40.2 to 54.8)	61.9 (48.2 to 73.1)	77.5 (56.2 to 91.2)
	NAR	54.2 (40.2 to 60.4)	44.2 (40.2 to 50.4)	61.8 (50.7 to 70.4)	79.4 (61.3 to 90.4)
*P* value		0.651	0.154	0.357	0.560
Isokinetic extension strength at 60°/s	AR	62.2 (52.6 to 69.4)	55.2 (48.6 to 62.6)	65.7(51.3 to 80.9)	76.3 (54.1 to 99.3)
	NAR	61.4 (54.0 to 68.7)	52.4 (46.0 to 61.7)	62.3 (52.1 to 76.7)	72.2 (58.1 to 91.7)
*P* value		0.521	0.542	0.405	0.268
Isometric flexion strength	AR	57.9 (46.4 to 65.5)	47.2 (42.3 to 55.5)	67.3(54.4 to 77.6)	87.4 (66.5 to 99.7)
	NAR	59.2 (50.7 to 66.1)	42.5 (40.6 to 54.1)	63.5 (51.5 to 76.6)	84.5 (62.4 to 99.1)
*P* value		0.741	0.311	0.264	0.212
Isometric extension strength	AR	51.4 (48.2 to 56.7)	54.1 (46.5 to 64.3)	69.8(54.8 to 81.8)	85.5 (63.2 to 99.4)
	NAR	58.2 (50.6 to 62.1)	51.6 (46.6 to 58.1)	67.5(52.6 to 78.6)	83.5 (58.7 to 99.1)
*P* value		0.424	0.154	0.121	0.186

Data are reported as median (range) in percentages

*LSI* limb symmetry index, *AR* anatomical reconstruction, *NAR* non-anatomical reconstruction

Postoperative clinical evaluations including Lachman test, pivot-shift test, KT-1000 arthrometer measurement, IKDC, Lysholm and Tegner scores showed no significant difference between the 2 groups (Table 4).

**Table 4:** Postoperative outcomes of knee stability and subjective knee function between the two groups at the final follow-up

	AR group	NAR group	*P* value
Lachman test, stable/I°/II°/III°	27/5/1/0	30/7/2/0	0.389
Pivot-shift test, stable/I°/II°/III°	27/3/3/0	32/6/1/0	0.421
KT-1000 side-to-side difference, mm	1.2 ± 2.1	1.4 ± 2.2	0.756
IKDC score	93.2 ± 13.2	95.2 ± 20.6	0.685
Lysholm score	92.4 ± 14.2	92.6 ± 8.4	0.951
Tegner score	8.3 ± 2.7	7.9 ± 2.3	0.141

*IKDC* International Knee Documentation Committee, *AR* anatomical reconstruction, *NAR* non-anatomical reconstruction

## Discussion

The most important finding of this study was that anatomical ACLR exhibited significant higher peak knee flexion torques under the velocity of both 180°/s and 60°/s, as well as isometric extension torque, as compared with non-anatomical ACLR at 6 months postoperatively, but no significant differences for all parameters were detected between the two groups at 12 months postoperatively. Also, both groups showed no significant difference in clinical outcomes regarding of knee stability and subjective knee function.

In our study, the results implied that anatomical ACLR showed a superior muscle strength as compared with non-anatomical ACLR at 6 months after surgery. However, there were no differences on muscle strength between the two groups at 12 months after surgery. The possible reasons may be as follows. Knees with non-anatomical ACLR potentially led to asymmetric knee kinematics and alteration of cartilage contact pattern [36, 37]. Yan et al. [38] compared anatomical and non-anatomic ACLR on gait kinematics with minimal 6-month follow-up, finding that operated knees with non-anatomical ACLR exhibited significant range of motion of anterior-posterior translation by approximately 0.5 cm than contralateral knees. Graft healing may be affected by tunnel position as well. Oshima et al. [39] reported low femoral tunnel was one of the factors significantly associated with high graft signal/noise quotient value, which indicated inferior graft healing. On the other hand, the study of Novaretti et al. [40] proved that deficit of quadriceps strength did not predict return to preinjury level of sport at 6 months postoperatively, which were consistent with the outcomes of 12 months after surgery in our study.

The LSI between the groups were not different regarding isokinetic strength at both 180°/s and 60°/s and isometric strength. To return the patient to ‘normal strength’ is an indicator of successful rehabilitation. ‘Normal’ limb symmetry index values are reported to be > 70–90 % [41, 42]. In our study, patients in both groups had an average LSI over 70 % at the final follow-up. Tegner score also showed the improvement of return to sports after ACLR. Iriuchishima et al. [43] evaluated muscle recovery after anatomical single-bundle ACLR, finding that at 12 months after surgery, average quadriceps strength was 85.1 ± 12.6 %, average hamstring strength was 96.7 ± 13.8 %. The results were similar with our study.

Measurement of knee muscles isokinetic and isometric torques after ACLR had been used in several studies. Iriuchishima et al. [43] evaluated peak flexion and extension isokinetic torque after anatomical single‑bundle ACLR using a quadriceps autograft, finding that average quadriceps strength and average hamstring strength were 85.1 and 96.7 %, respectively, at 12 months after the surgery. Martin-Alguacil et al. [16] performed a randomized controlled trial to compare peak isokinetic torques after ACLR with quadriceps tendon (QT) versus hamstring tendon (HT) autografts. They found that the HT group showed a greater increase in peak torque in extension than the QT group did at 60°/s, 180°/s, and 300°/s. Czamara et al. [22] used isometric and isokinetic test to monitor and assess the outcome of physiotherapy for patients after ACLR, believing that there were persistent torque deficits of injured knees after 17-week postoperative physiotherapy.

Strength recovery after ACLR is of great importance for patients who want to return to sport, especially athletes [16, 17]. Muscle strength may also have correlations with knee function. Wang et al. [44] follow 88 patients who underwent double-bundle hamstring ACLR and performed a second-look arthroscopy at an average of 24 month postoperatively, finding that greater than 80 % recovery of quadriceps strength after ACLR is associated with less severe patellar cartilage damage. In the study of Palmieri-Smith et al. [45], 73 patients were tested at the time they were cleared for return to activity after ACLR. The results indicated that patients with high and moderate quadriceps strength symmetry had larger central activation ratios as well as greater limb symmetry indices on the hop for distance compared with patients with low quadriceps strength symmetry. Similarly, knee flexion angle and external moment symmetry were higher in the patients with high and moderate quadriceps symmetry compared with those with low symmetry. However, Thomeé et al. [46] believed that muscle function tests were not demanding enough or not sensitive enough to identify differences between injured and non-injured sides. More studies with long-term follow-up are required to validate the influence of muscle strength after ACLR.

Tunnel preparation is the most important procedure in ACLR. For the tibial side, it is more consistent because tibial tunnel aperture has multiple reference points, e.g., edge of anterior horn of lateral meniscus, medial intercondylar eminence of the tibia and remanent ACL tissue. However, in femoral side it is more variable. According to a multicenter study with the largest collected data of ACL revision, the malposition of the tunnel socket accounts for most of technique errors, which are the main cause of atraumatic ACLR failure [47]. Femoral tunnel malposition is 3 times more frequent than tibial tunnel malposition [48]. In our study, the femoral tunnel position was measured with the use of Bernard quadrant method, which was applied in several studies [6, 39]. The anatomic position of the femoral tunnel socket for single-bundle ACLR is defined in line with the study of Xu et al. [29]. They systemically reviewed 13 studies of the ACL femoral footprint position and combined data, concluding that the standard area of femoral footprint of the ACL as a whole bundle is a circle with a center of 27.53 %, 35.85 % (x, y), and a radius of 4.58 %, 9.2 % (x, y), respectively. However, in this study, about 54.17 % (39/72) included patients had non-anatomical femoral tunnel position. We used edge of cartilage and clock method to locate femoral tunnel, which might lead to variety of distribution. Literature reported the rate of non-anatomical femoral tunnel position after ACLR with AM drilling technique ranged from 61.76 to 73 %[49]. Compared with tibial tunnel, it is more difficult to locate femoral tunnel at the anatomical position, as femoral footprint of ACL vary in patients with different conditions, e.g., gender, BMI and injury time [50–52].

The current study has several limitations. First, the sample size was small, and the length of follow-up was relative short, which limited assessment on long-term complications and secondary treatment. Second, reported standard area for anatomical ACL footprint rather than the contralateral normal ACL footprint was used for the determination of tunnel placement. Third, only knee flexion and extension torques at velocities of 60°/s and 180°/s were studied. Advanced Isokinetic test under different movements of knee and velocities should be further evaluated. In addition, the study failed to randomize the groups initially as the grouping was performed after the surgery, which increased the confounding risk of patient selection. Lastly, tibial tunnel position, graft sizes, conditions of meniscal injuries and their treatment manners may have affected the outcomes as well.

## Conclusions

In conclusion, this study revealed that the position of femoral tunnel aperture of ACLR was associated with recovery of hamstring and quadriceps strength. Compared with non-anatomical ACLR, anatomical ACLR showed a better hamstring and quadriceps strength at 6 months postoperatively. However, the discrepancy on hamstring and quadriceps strength between the two groups vanished at 1 year postoperatively.

## Data Availability

The datasets used and/or analyzed during the current study available from the corresponding author on reasonable request.

## Abbreviations

ACL: Anterior cruciate ligament
ACLR: Anterior cruciate ligament reconstruction
TT: Transtibial
AM: Anteromedial
AL: Anterolateral
IKDC: International Knee Documentation Committee
AR: Anatomical reconstruction
NAR: Non-anatomical reconstruction
